# Would You Tell Everyone This? Facebook Conversations as Health Promotion Interventions

**DOI:** 10.2196/jmir.3231

**Published:** 2014-04-11

**Authors:** Jonathan Syred, Carla Naidoo, Sarah C Woodhall, Paula Baraitser

**Affiliations:** ^1^HIV & Sexual Health Research GroupKing's College LondonLondonUnited Kingdom; ^2^National Chlamydia Screening ProgrammePublic Health EnglandLondonUnited Kingdom; ^3^HIV & STI DepartmentPublic Health EnglandLondonUnited Kingdom; ^4^Department of Sexual Health and HIVKings College Hospital NHS Foundation TrustLondonUnited Kingdom

**Keywords:** social media, chlamydia, health promotion

## Abstract

**Background:**

Health promotion interventions on social networking sites can communicate individually tailored content to a large audience. User-generated content helps to maximize engagement, but health promotion websites have had variable success in supporting user engagement.

**Objective:**

The aim of our study was to examine which elements of moderator and participant behavior stimulated and maintained interaction with a sexual health promotion site on Facebook.

**Methods:**

We examined the pattern and content of posts on a Facebook page. Google analytics was used to describe the number of people using the page and viewing patterns. A qualitative, thematic approach was used to analyze content.

**Results:**

During the study period (January 18, 2010, to June 27, 2010), 576 users interacted 888 times with the site through 508 posts and 380 comments with 93% of content generated by users. The user-generated conversation continued while new participants were driven to the site by advertising, but interaction with the site ceased rapidly after the advertising stopped. Conversations covered key issues on chlamydia and chlamydia testing. Users endorsed testing, celebrated their negative results, and modified and questioned key messages. There was variation in user approach to the site from sharing of personal experience and requesting help to joking about sexually transmitted infection. The moderator voice was reactive, unengaged, tolerant, simplistic, and was professional in tone. There was no change in the moderator approach throughout the period studied.

**Conclusions:**

Our findings suggest this health promotion site provided a space for single user posts but not a self-sustaining conversation. Possible explanations for this include little new content from the moderator, a definition of content too narrow to hold the interest of participants, and limited responsiveness to user needs. Implications for health promotion practice include the need to consider a life cycle approach to online community development for health promotion and the need for a developing moderator strategy to reflect this. This strategy should reflect two facets of moderation for online health promotion interventions: (1) unengaged and professional oversight to provide a safe space for discussion and to maintain information quality, and (2) a more engaged and interactive presence designed to maintain interest that generates new material for discussion and is responsive to user requests.

## Introduction

### Background

Health promotion interventions on social networking sites (SNS) harness user-generated content and the power of networks to improve health [1]. They reach large numbers of people and have the capacity to communicate tailored messages quickly [2]. Stimulating user-generated content is important to the success of this approach because interactivity influences reach with new participants joining the conversation as they observe the activity of their online friends [3]. Observing or participating in such conversations shares information on the beliefs and experience of peers, an important influence on behavior [4], and interaction with health promotion information, (commenting, challenging, modifying) facilitates engagement with this material and therefore learning from it [5]. User-generated content personalizes generic health promotion messages and adapts them for specific populations, increasing their relevance to new audiences [6].

Although user-generated content has important advantages, it may also lead to widespread sharing of poor quality information and material that is offensive to individuals or groups [6,7]. As sharing of personal experience is an integral part of online community activity, SNS health promotion interventions carry risks for participants. These risks are potentially high where they involve sharing of sexual health information between young people [8], and interventions of this sort require careful moderation to prevent harmful activity.

Although the interactivity of SNS health promotion interventions is an important element of their capacity to deliver health promotion messages, they have been variably successful in stimulating and/or supporting user-generated content and many remain inactive [9]. Levels of interactivity are likely to reflect individual or group motivations for engaging with the site, the technology required to access it, the relationships formed within the group, and the structures for interaction including the presence of a moderator and moderator behavior [10-12]. The role of the moderator in health promotion campaigns on SNS are not fully understood [13], with a perceived trade-off being between encouraging activity and retaining control of content.

A recent systematic review of the use of SNS for health promotion identified a lack of evidence on effective approaches to stimulating interaction [9]. Although there is significant literature on interactions within online communities, particularly those that are business oriented, there is less research in relation to health promotion interventions.

The key metrics to understand interactions on social networking sites are the number of posts and their content [12]. To understand the factors that facilitate interaction on SNS for health promotion, we looked at the volume, pattern, and content of interaction within a national health promotion campaign using the SNS site, Facebook. The health campaign, “Say Yes to the Test”, aimed to encourage testing for genital chlamydia infection among young people in England between January and June 2010 by generating discussion on chlamydia and chlamydia testing. The SNS page was one element of a larger multimedia campaign by the English Department of Health and the Health Protection Agency (now Public Health England) entitled “Sex Worth Talking About”.

We collected quantitative data on the volume of interaction and qualitative data on its content. We sought to identify and study what stimulated and maintained interaction on this Facebook-based sexual health promotion campaign with particular attention to the role of the moderator in stimulating interaction and the role of users in modifying messages. Our research question was “What elements of moderator and participant behavior stimulated and maintained interaction with a sexual health promotion site on Facebook?”

### Setting and Approach

*Chlamydia trachomatis* is the most commonly diagnosed bacterial sexually transmitted infection in the United Kingdom. The prevalence is highest among young people aged 15-24 and infection is usually asymptomatic [14]. The English National Chlamydia Screening Programme aims to offer all sexually active young people under the age of 25 years testing for this infection annually or on change of sexual partner [15].

The “Chlamydia Worth Talking About” strand of the “Sex Worth Talking About” Campaign was a multimedia campaign launched in January 2010 that aimed to encourage (1) open discussion about chlamydia and (2) acceptance of a chlamydia test when offered by a health professional.

The campaign included television, radio, and billboard advertisements and a Facebook page that encouraged discussion about chlamydia testing, entitled “Say Yes to the Test”. The Facebook site was promoted via a separate digital media strategy. The target audience for the campaign was young people (male and female) in England aged 15-24 years. Advertisements (traffic drivers) placed on sites used by young people suggested either that chlamydia “knew” someone they knew or that it had “poked” them. It was anticipated that curiosity arising from these ads would cause the user to click on them. The welcome page for the health promotion intervention offered options to become a fan of the page and post comments on the “comment wall”. This gave users the opportunity to affirm their positive attitude among their own friends, spreading the word and encouraging conversations.

The moderation style employed on the “Say Yes to the Test” site was non-interventionist. Only comments that were completely unrelated to sexual health or that were directly offensive were removed. Factually incorrect comments were left on the site giving the peer group opportunity to respond before the moderation team intervened.

### Facebook Environment

A Facebook community page is organized around a publically displayed wall where new content (messages, media, or links) can be added by the owner (or moderator) and other Facebook users [16]. Any form of interaction, whether liking or posting content, can be seen on the user’s newsfeed. This may be shared automatically with people within their network depending on the level of interaction that they have specified with the other user [17].

## Methods

The literature on online communities suggests that the key metrics for evaluation of this type of intervention are the volume of member contributions and the quality of the online relationships formed [12]. We therefore used a combination of quantitative data to describe the volume of contributions and completed a qualitative analysis of the content of the interaction. We described the users and their patterns of use and analyzed the text posted. We looked at data from the first 5 months of the campaign from January 18 to June 27, 2010, as this was the period of highest activity on the “Say Yes to the Test” site. The total number of fans, wall posts, and comments over time, fan demographics (gender, age, and country where page was accessed) were obtained from the Facebook page administrators. Google analytics were used to document numbers using the site and viewing patterns. We captured the page content from within the study period using the NCapture function of NVivo 10 software and used simple counting to describe user and moderator content, discussion thread patterns, and moderator intervention.

The qualitative analysis was completed by 2 investigators (JS and PB) using the framework approach and initially coding together to ensure consistency. Disagreements were discussed until a consensus was reached. All coding was done using NVivo 10.

We completed an initial process of familiarizing ourselves with the data by reading and re-reading the posts, and from this, we identified an initial set of themes to describe interaction with the site: (1) patterns of posting, and (2) content of posts. From this, we developed three coding strategies each building on the previous one (Table 1). We kept the two main elements of coding—patterns and content—constant.

We applied for ethical approval for this study from a local National Health Service Research Ethics Committee. The committee reported that ethical approval was not required since our analysis was of publically available data that we did not link to personal profiles. Our use of the data is consistent with guidelines on the ethical conduct of qualitative research on online communities [18]. Furthermore, we have followed recent recommendations from research in similar contexts and used non-verbatim quotes to prevent identification of the users through a search engine [19]. These were constructed through interchanging the words from several posts from different authors that were thematically similar.

**Table 1:** Coding categories.

	Patterns of posting	Content of posts
Round 1 coding: simple description of patterns and content of interaction (content codes)	Conversation length: conversations coded by number of posts	Attitudes to chlamydia/testing
		Experience of testing
		Offensive/stigmatizing/inaccurate material
	Conversation source: content coded by source of posts (users/moderator)	Requests for information/advice
Round 2 coding: factors that triggered interactivity (response codes)	Conversation length: factors associated with longer conversation length	Responses to inaccurate information, challenges to key messages or stigmatizing or offensive material
	Conversation source: factors that triggered user or moderator intervention	Responses to key messages (eg, questioning/endorsing)
Round 3 coding: responses to initial interactions	Interactivity: patterns of questions and answers between users and other users, and users and the moderator.	Content of unresolved and complex issues and moderator responses to these

## Results

### Patterns of Interaction

There were 191,072 page views during the 5-month study period. The largest cumulative total number of fans was 68,174 fans at Week 7. Two-thirds (64%) of the fans were female, 96% of fans were aged 13-24 years (Table 2), and 95% of fans were from the United Kingdom.

**Figure 1:**
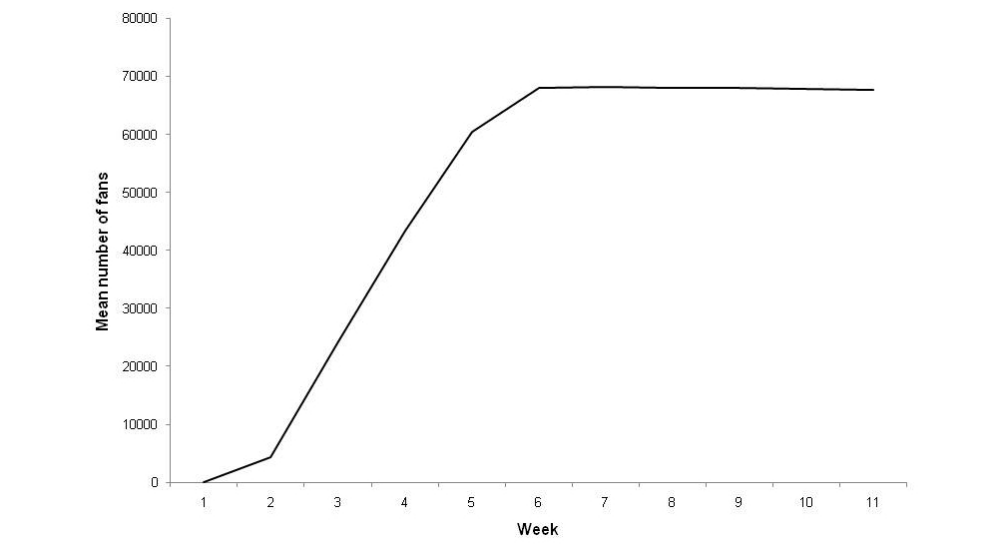
Total fans, Weeks 1 (18/01/2010) to 10.

**Figure 2:**
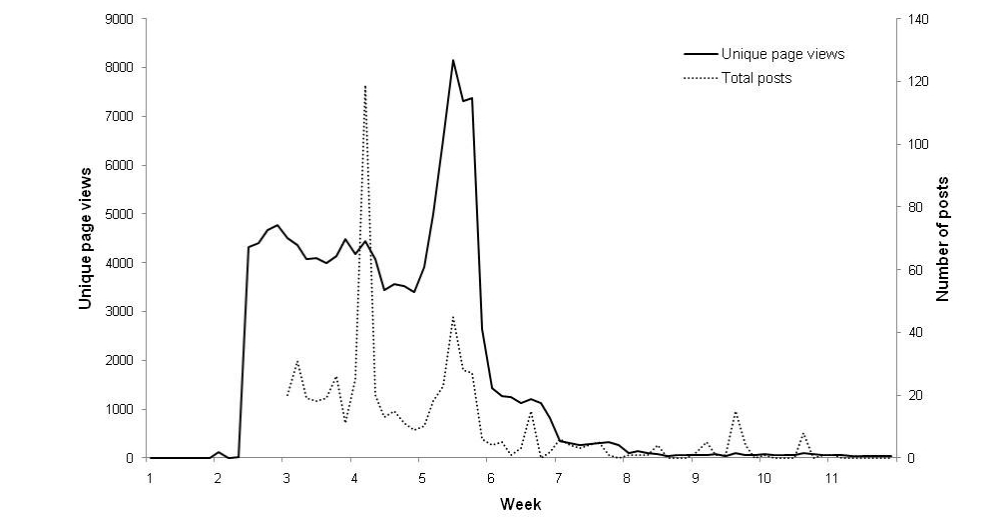
Total wall posts/comments and unique page views, Weeks 1(18/01/2010) to 10.

The number of fans increased from the launch of the site until Week 7, when the advertising ceased and when the total number of fans accumulated during this 7-week period reached 68,174 (6/3/2010). It subsequently decreased by about 100 fans per week (Figure 1). The majority of the activity was seen between Weeks 2 and 6 (Figure 2).

We collected 888 interactions (508 posts, 380 comments) from 576 unique users; 93% of content was from users. In contrast to the 68,174 “likes” for the site, content interaction received a much smaller number with 483 “likes” for 156 posts or comments.

Most users interacted once (77.6%) and fewer than 2% posted more than five times, with 17 posts the highest number of interactions from a single user.

There were 164 threads where one or more users commented on an original post. These were usually short (one comment followed by one post), although a minority were longer with the longest being made up of 56 comments.

The number of fans and posts related closely to the advertising campaign and activity decreased very rapidly once the advertising stopped, suggesting that the interactions on the site themselves were insufficient to maintain user input or to generate new participants.

**Table 2:** Age of fans of “Say yes to the test” by sex.^a^

Age range	Male, %	Female, %	All, %
13-17	43	54	50
18-24	54	43	47
25-34	2	2	2
35-44	<1	<1	<1
55+	<1	<1	<1

^a^The proportion in each age group calculated on a daily basis and averaged across the data collection period.

### Content of Interaction

#### Overview

Conversations covered key issues on chlamydia and chlamydia testing including the lack of symptoms, consequences of infection, and experience of getting tested and treated.

#### Attitudes to Chlamydia Testing

Posts endorsed chlamydia testing as the right thing to do, often reproducing the campaign message “Say Yes to the Test”. Sixty-two posts encouraged others to get tested for peace of mind because it is easy to do and to prevent subfertility:

Bout time peeps startd tikin about this…y not go gt check 4eurything its 20mins out ur life…tht ultimately cud save ur life and tht of ur future children and new partners...sense...people..gud on ya if ya dne it...an if u ant y…x

Poster 1

Although testing is generally presented as the “right thing to do”, there was disagreement about its implications. Some participants felt that testing suggests risk of infection through multiple partners with either negative implications (promiscuity) or positive ones (sexual experience). Others suggested that it signals irresponsible behavior (no condoms used), carelessness (poor condom use), or bad luck. Some felt that it is a routine part of looking after yourself:

People who say like “oh im not ashamed, I have this test all the time”...stop having sex with so many diff men, then you wouldn’t need to have it done all the time =]

It’s making it “shameful” that prevents people from going and getting tested. The skanky ones are the ones that don’t get tested you fool. If anything everyone that is a fan of this group are the absolute opposite.

I went for it its all good. I got the all clear too. just go on a random moment, just means your looking after yourself

#### Uncertainty Around Testing Messages

User-generated content identified and did not resolve uncertainty around some aspects of chlamydia testing. This maintained conversations by generating disagreement and encouraged sharing personal experience. It identified questions that were important to participants but failed to resolve them because the approach to moderation was simple, factual responses without engaging in conversation.

The effectiveness of condoms in preventing chlamydia was an example of this and a subject of concern. If condoms protect against chlamydia, why should those who use condoms consistently still be tested? Similarly, users were confused about the risk of subfertility following chlamydia infection. Some posters reported that after a single episode of infection becoming pregnant was impossible and that chlamydia infection could therefore be used as a method of contraception.

None of these questions have simple answers since the effectiveness of condoms depends on how consistently and carefully they are used. The impact of chlamydia on subfertility is important but contested. It is very difficult to measure accurately and not completely resolved by treatment.

the result was.....positive. no kids for me shammmme init

Poster 1

oviously twat

Poster 2

u cn still have kids if u get it treated

Poster 3

#### Results of Getting Tested

Most of those who discuss their experience of testing report their results as negative and celebrate this (71/128) with “I’m clean” (63/128) and “never thought I had it and now I know I don’t” as common posts associated with a tangible sense of relief after the anxiety of waiting for results.

Many fewer comments (28/128) report a positive chlamydia test. Most of these fall into the “just joking” category, often emphasizing “I don’t care” or “I’m pleased” or exaggerating the consequences, for example, “my penis will drop off”. The minority of posts that asked for help were important opportunities for participants to offer support and challenge stigma:

im really worried because I think I have chlamydia, … WHAT SHOULD I DO?!

Poster 1

have u got any symptoms like a rash or anything like urination being different ive added u ive had it b4 ill chat to u if u like im xxxxxx unlike some idiots on here

Poster 2

There were marked differences in approach to the topic between those who were seriously concerned about chlamydia, from a personal or public health perspective, and those who used the topic as an opportunity to post in a joking manner.

#### Modification and Repetition of Health Promotion Messages

Many posts and comments (n=158) contained messages that adapted but were consistent with key campaign messages about testing and condom use, for example, “dont be fool wrap your tool, strap up before you whack up boys”.

Whether to share personal information in a public forum was discussed referencing both the need for openness to provide information and support and the personal consequences of sharing information online where it is accessible by both those from offline social networks and strangers:

had it, got rid of it, get tested few times a year just coz I can

Poster 1

why wud u tell everyone this?? Nice lass u r eh!! well I see u deleted ur comment nd I mean why wud u want everyone to know u had chlamydia twice?? Just makes u look like a skank tbh them things are usually kept private

Poster 2

#### Harmful or Offensive Messages

There were 39 posts classified as potentially harmful or embarrassing, as a named individual or poster of a previous message was the subject of offensive language or inappropriate remarks. We were unable to establish the tone of many of the challenging messages without further context including knowledge of the relationship between posters, for example, “I got chlamydia off Stevey”. Some harmful posts were ignored by the moderator to discourage continued posting. We were unable to view those deleted with a private warning message. Of all 164 comment threads, harmful or offensive comments appeared in 31 (18.9%) of them at least once. Of these message chains, 40 had four or more comments following an original post. Of these 40 longer threads, offensive content appeared in 12 (30%) at least one or more times. We had hypothesized that potentially harmful content or challenges of key messages might stimulate message chains, but this content did not appear to drive longer threads.

### The Role of the Moderator

Of all 888 posts and comments, 62 (6.9%) were by the moderator. The approach adopted was to choose from a list of standardized responses and repeat these as the same issue occurred. There was no attempt to engage in the conversation, stimulate discussion, or reproduce the language used by posters. This means that the tone of posts by the moderator was very different from that of the users. The moderator did not respond to misinformation posted on the site immediately but allowed time for other posters to correct it and then endorsed the corrections:

Hi xxx! Just to clarify, neither chlamydia infection or the health consequences of untreated chlamydia infection would lead to penis amputation! However, untreated chlamydia can cause painful inflammation in one or both testicles… To find out how it is important to “Say yes to the test” log on to….

Of the moderator’s interactions, 53/62 (85%) were a response to an existing conversation. Of these comments, 43/53 (81%) were the last or second to last comment in the thread.

Users appreciated moderator comments with 192 likes (40%) for moderator interactions. The highest number of likes for a user interaction was 10 and for a moderator interaction 63. There were no cases where the posters expressed dissatisfaction with comments from the moderator.

A total of 80% (51/64) posts or comments that challenged campaign messages or contained inaccurate information or questions were corrected, with 47 (73%) responses within four posts, that is, appearing in the same window as the post on Facebook newsfeeds. Over half of these responses came from users:

where you get free test from??

Poster 1

from the docs just go and ask for one or go sex health clinc x

Poster 2

boots do them for free aswell x

Poster 3

any boots?

Poster 1

You can order one online. Its great. You get a pippette :D

Poster 4

i got mine from school, they were giving them out

Poster 5

## Discussion

### Principal Findings

We aimed to study what stimulated and maintained interaction on a sexual health promotion site on Facebook with particular emphasis on the role of the moderator. The stated aim of the site studied was to promote chlamydia testing, to reinforce key messages about chlamydia infection, and to promote the attitudes that make testing more likely [20]. The potential outcomes of participation included information exchange, social support and social interaction [12], as well as better sexual health through increased likelihood of chlamydia testing. The potential risks of participation include the consequences of sharing personal information online and exposure to harmful online behavior such as the posting of inflammatory, hostile, or insulting behavior.

The “Say Yes” site attracted a very large number of potential users, but there was no evidence of sustained interaction. Single visits to the site could have provided information on testing but more active engagement with the site (repeat visits or posting) would be more likely to change attitudes [4]. More sustained interaction from a significant number of people would be associated with the development of an online community, either self-sustaining or supported by new stimuli for discussion from the moderator. One definition of an online community is “a collective group of entities, individuals or organizations that come together either temporarily or permanently through an electronic medium to interact in a common problem or interest space” [21]. The idea of a shared area of interest is important for our discussion. If online communities must make a transition from early engagement to self-sustaining interaction [22] and if key metrics to monitor the success of online communities include the volume of member contributions and the quality of the online relationships formed [12], our analysis suggests that this intervention was not successful in making the transition to a mature online community. There was a low volume of posts once the advertising ceased and the development of superficial relationships as evidenced by the short discussion threads and lack of continued engagement.

Major influences on the amount and type of interaction with SNS-based health promotion interventions include the structure of relationships between users [10], the role of the moderator [11], and the content of the online discussion [23]. Drawing on this work, we propose that barriers to sustained interaction could include an audience that did not have a sufficiently shared approach to this topic, a lack of new content from the moderator, a definition of content that was too narrow to hold the interest of participants, and a lack of moderator responsiveness to participant needs. The evidence for these proposals from our study are explored below.

On the “Say Yes” site, there was some indication of shared interest in chlamydia as users had “liked” the site and there was reference to a common experience of chlamydia testing among some of those who posted. People join online discussions where they identify others with similar interests and viewpoints [21,24]. An effective online community will attract users with sufficient common interest to provide a safe space for discussion and sufficient difference to provide material for conversation. Our findings suggest marked differences in approach to the topic, for example wide differences in how seriously they approached the content with some users sharing personal experience of and concerns about chlamydia infection while others ridicule the idea of sexually transmitted infection. Since support from others is an important incentive to stay engaged with online communities [24,25], the lack of a supportive environment from some users might have limited the willingness of those who did post topics for discussion to pursue their interest in this forum.

We think that the nature of the subject matter, a mainly short-term condition that is easily treated, meant that the incentives to stay engaged with the site are very different from those that support users with long-term conditions. Strategies to maintain engagement could have included broadening the discussion to other areas of sexual health over time so that new content was generated for discussion, providing a greater incentive to stay engaged and a promise of more information on new, but related topics.

The approach to moderation was reactive (it did not generate new material), unengaged (it provided simple factual responses), tolerant (it gave participants time to respond to inaccurate or challenging material), simplistic (it ignored controversial or complex material and repeated simple health promotion messages), and professional in tone. This fulfilled the important function of maintaining the quality of information on the site [6] and provided space for user-generated content, but it was also repetitive and ultimately uninteresting and did not provide sufficient encouragement for interaction at this early stage of online community development. In particular, it did not respond directly to the needs of users, other than with repetitive health promotion messages and it was not part of a strategy that acknowledged the changing role of the moderator as an online community develops [12]. The idea that online communities progress from small groups with a common interest and informal rules to larger and more established ones with more formal rules and high levels of participation is well documented [12,22]. Groups of the sort that we studied that are created by advertising for a purpose identified by an external agency may follow a different pattern of development closer to online communities developed for consumers by businesses (eg, [26]). Here the early stages of online community development are described slightly differently with an “on board” stage where the community is highly dependent on founder participation and the nature of participation is unclear, to an “established” stage where relationships within the membership base have been established and are less reliant on founder participation.

Delaying response was important to provide space for user-generated content but also carries risks. Users answered almost all of the questions posed by other users but corrected only a third of the inaccurate information posted. With only the last four comments of a discussion thread appearing on Facebook newsfeeds, inaccurate content should be corrected within three comments but this risks closing down discussions. Leaving material that is potentially harmful to a named individual is dangerous [8,27-29] and, from our analysis, is not associated with stimulating conversation. Although removal of this material could encourage repeated posting, we recommend that it be removed. While personally offensive responses were removed, the moderator could not respond to private offensive messages on individual Facebook pages. More information is needed to quantify the risk to participants of sharing information in this way and the responsibilities of the public health agencies who run this type of intervention.

We did not set out to measure the impact of the intervention. The impact of the whole media campaign (of which this intervention was one element) on chlamydia testing rates has been evaluated and shown to be associated with an increased number of positive tests linked to increased testing of high risk individuals although not an increased number of tests overall [25]. We do not know what impact, if any, was related specifically to the SNS intervention; however, the site did have some characteristics of an effective health promotion intervention. Those who visited the site and reviewed the conversation would have received correct information on chlamydia, information on attitudes to chlamydia and chlamydia testing among their peers including normalization of chlamydia testing, reassurance that chlamydia can be easily treated, signposting to additional sources of information, and promotion of condoms to prevent chlamydia infection. They would also have had an opportunity to actively engage with this material, modifying and personalizing it—both are activities associated with active learning and effective health promotion interventions [5].

### Limitations

Our findings are limited by our lack of data on those who participated on this site. Without this, we do not know how they are similar or different from the rest of the population targeted by this campaign. Similarly, we have no data on interactions with the site that did not involve posting, for example, we do not know how long each participant spent on the site, what material they read or whether they returned to the site, except where they returned to the site to post. In addition, we were not able to obtain data on posts that were removed by the moderator or warnings sent to individual participants. Finally, we have no data on the impact of this SNS campaign on the knowledge, attitudes, and behaviors of those who viewed or participated in this intervention.

### Conclusions

Our findings suggest this health promotion site provided a space for single user posts but not a self-sustaining conversation. Possible explanations for this include little new content from the moderator, a definition of content that was too narrow to hold the interest of participants, and limited responsiveness to user needs.

Implications for health promotion practice include the need to consider a life cycle approach to online community development for health promotion and the need for a developing moderator strategy to reflect this. This strategy should reflect two facets of moderation for online health promotion interventions: (1) unengaged and professional oversight to provide a safe space for discussion and to maintain information quality and (2) a more engaged and interactive presence designed to maintain interest that generates new material for discussion and is responsive to user requests.

## Abbreviations

SNS: social networking site
